# Coercivity Mechanism of (Nd_0.8_Ce_0.2_)_2.4_Fe_12_Co_2_B Ribbons with Ferromagnetic Grain Boundary Phase

**DOI:** 10.3390/ma10091062

**Published:** 2017-09-11

**Authors:** Heyun Li, Yang Liang, Xiaohua Tan, Hui Xu, Pengfei Hu, Kezhi Ren

**Affiliations:** Institute of Materials, School of Materials Science and Engineering, Shanghai University, Shanghai 200072, China; liheyunde@126.com (H.L.); powder_material@163.com (Y.L.); huixu8888@shu.edu.cn (H.X.); kezhiren0920@163.com (K.R.)

**Keywords:** permanent alloys, magnetic field annealing treatment, ferromagnetic grain boundary phase, coercivity mechanism

## Abstract

Understanding the coercivity mechanism has had a substantial impact on developing economically more attractive RE-based (RE = rare earth) permanent materials because of price volatility of key RE metals (i.e., Nd and Dy) in recent years. In this work, we investigated the microstructure and magnetic properties of melt-spun (Nd_0.8_Ce_0.2_)_2.4_Fe_12_Co_2_B ribbons and annealed samples at 773 K for 15 min with 1 Tesla (T) magnetic field to better understand the coercivity mechanism. We found hard magnetic grains were surrounded by thin and continuous layers along the grain boundaries (GBs) with a high concentration of ferromagnetic elements (Fe + Co >74 at%). The obvious positive peak in the *δM* plot and the interaction domain structure observed by Lorentz magnetic microscopy indicate that there is strong exchange coupling interaction through the ferromagnetic GB phase between hard magnetic grains. The annealing in an applied magnetic field of 1 T increases the remanence by enhancing the exchange coupling interaction, leading to a maximum product energy ((*BH*)*_max_*) which is 16% higher than that of melt-spun ribbons. We also studied the temperature dependence of the coercivity in a temperature range of 300–500 K, and proposed that the coercivity of melt-spun (Nd_0.8_Ce_0.2_)_2.4_Fe_12_Co_2_B ribbons with ferromagnetic GB phase at room temperature was from the combination of strong domain-wall pinning and nucleation. The same mechanism works in the annealed ribbons.

## 1. Introduction

Nd_2_Fe_14_B based permanent magnets (PMs) have attracted considerable research interest since their discovery in 1984, and are widely used in various fields including electronic, hybrid electric vehicles, and wind turbines because of their excellent magnetic properties [1,2,3]. Melt-spun Nd-Fe-B ribbons consist of randomly oriented Nd_2_Fe_14_B grains with an average diameter of less than 100 nm. The powders pulverized from melt-spun Nd-Fe-B ribbons are currently used as raw materials for the production of bonded magnets and hot-pressed magnets [4]. In recent years, there has been great effort in developing economically more attractive RE-based PMs (RE = rare earth) because of the limited natural resources and high cost of key RE metals (i.e., Nd and Dy) [5,6]. The element cerium (Ce), would be one of the suitable elements to form alloys by partially substituting Nd because of the earth abundance and fairly low cost [7,8,9]. However, substitution of Ce for Nd in Nd_2_Fe_14_B alloy deteriorates the hard magnetic properties because the magnetic properties of Ce_2_Fe_14_B are inferior to those of Nd_2_Fe_14_B [4]. In 2015, Pathak et al., reported an unexpected increase of the coercivity (1409 A∙m^−1^) and (*BH*)*_max_* (100 kJ∙m^−3^) for melt-spun (Nd_0.8_Ce_0.2_)_2.4_Fe_12_Co_2_B ribbons by simultaneous substitution of Nd by Ce, and Fe by Co [10]. The segregation of heavy elements was observed along the grain boundaries (GBs), which probably accounts for the excellent magnetic properties. However, the coercivity mechanism has still not been established.

Coercivity is an extrinsic magnetic property and it is intimately related to the microstructure of a magnetic material. In Nd_2_Fe_14_B based alloys, the GB phase has a strong influence on the coercivity. For example, the coercivity is enhanced by isolating Nd_2_Fe_14_B grains with non-ferromagnetic Nd-rich phase at the GBs [11,12,13,14]. In contrast, the GB phase containing large fractions of Fe and Co (Fe + Co >65 at%) may be ferromagnetic, which leads to exchange coupling between Nd_2_Fe_14_B grains and reduces the coercivity [15,16,17,18,19]. Hence, the coercivity mechanism of Nd_2_Fe_14_B based alloys with GB phase needs to be further investigated. Remanence is another extrinsic magnetic property and can be improved by grain size refinement or an enhancement of the exchange coupling interaction using magnetic field annealing heat-treatment [20,21,22]. In this study, we investigated the microstructure and magnetic properties of melt-spun (Nd_0.8_Ce_0.2_)_2.4_Fe_12_Co_2_B ribbons as well as samples annealed in a 1 T magnetic field to obtain a deeper insight into the coercivity mechanism. Moreover, we used magnetic field annealing treatment to modify the chemical composition of the GB phase to achieve a relatively high level of coercivity and remanence, and obtained high performance permanent materials. We found that the GB phase had high concentration of ferromagnetic elements (Fe + Co > 74 at%) in melt-spun ribbons and annealed samples at 773 K for 15 min with 1 T magnetic field. The remanence of annealed samples was enhanced due to strengthened exchange coupling interaction through the ferromagnetic GB phase, leading to 16% higher (*BH*)*_max_* than that of melt-spun ribbons. Moreover, we proposed the coercivity in melt-spun (Nd_0.8_Ce_0.2_)_2.4_Fe_12_Co_2_B ribbons with ferromagnetic GB phase at room temperature was from the combination of strong domain-wall pinning and nucleation.

## 2. Materials and Methods

Ingots with stoichiometric composition (Nd_0.8_Ce_0.2_)_2.4_Fe_12_Co_2_B were prepared by arc-melting the mixture of pure metals Nd (99.99%), Fe (99.99%), Co (99.99%), Ce (99.99%), and Fe-B alloy in an argon atmosphere. Ingots were re-melted four times for homogenization. Ribbons were obtained by melt-spinning in the argon atmosphere at a wheel speed of 15 m∙s^−1^. During melt-spinning, the distance between an orifice of a quartz crucible and the copper wheel surface was maintained at 8 mm. The quenching temperature and chamber pressure were maintained at 1588 K ± 5 K and 0.05 MPa. The heat-treatments were carried out for melt-spun ribbons in the range of 573–1023 K for 15 min using a vacuum furnace with 1 T magnetic field. The direction of the magnetic field was parallel to the longitudinal ribbon plane. The density of the (Nd_0.8_Ce_0.2_)_2.4_Fe_12_Co_2_B alloy was 7.71 g∙cm^−3^ using Archimedes principle. The exchange interaction curve (*δ*M-H curve) was determined from the measurement of isothermal remanence magnetization (IRM) and DC demagnetization (DCD) curve. The sample for the IRM curve was virgin state. The detailed measurement of the IRM and DCD curve was introduced in reference [23]. The magnetic property at room temperature and the temperature dependence of the coercivity in the range of 300–500 K were measured by a Physical Property Measurement System (PPMS) (Quantum Design, San Diego, CA, USA) equipped with a 9 T magnet. The magnetic domain structure was investigated by Lorentz microscopy using JEM-2100F (JEOL Ltd., Akishima, Tokyo, Japan) transmission electron microscope (TEM) operating at 200 kV. X-ray diffraction (XRD) patterns were recorded using a D/max-2550 diffractometer (Rigaku Corporation, Akishima-Shi, Tokyo, Japan) with Cu K*α* radiation. The high angle annular dark field (HAADF) image and elemental characterization were performed by a scanning transmission electron microscope with energy-dispersive X-ray spectroscopy (STEM-EDS) (JEM-2100F, JEOL Ltd., Akishima, Tokyo, Japan). Cross-section samples near the wheel surface (that is, the surface in contact with the copper wheel) and free surface of ribbons for TEM and atom probe tomography (APT) observations were made by a Helios 600i focus ion beam (FIB) (FEI Corporate, Hillsboro, OR, USA). The APT characterizations were carried out in a CAMECA Instruments LEAP4000X-HR local electrode atom probe (Ametek Inc, Berwyn, PA, USA). Data acquisition was performed at a specimen tip at 50 K with a target evaporation rate of 0.5%, and a pulse-to-standing DC voltage ratio of 20%. Atom probe data reconstruction was conducted using the CAMECA Integrated Visualization and Analysis Software (IVAS^TM^ 3.6.8).

## 3. Results

**Figure 1:**
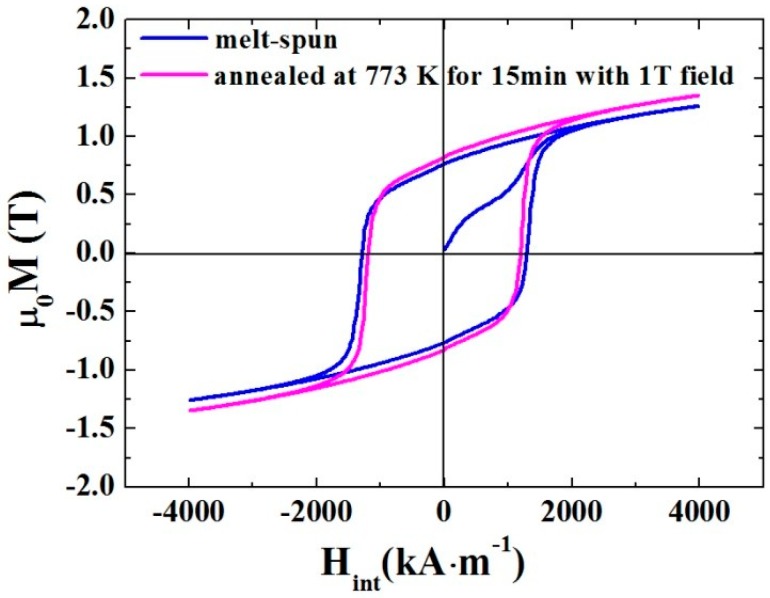
Hysteresis loops of melt-spun (Nd_0.8_Ce_0.2_)_2.4_Fe_12_Co_2_B sample and the sample annealed at 773 K for 15 min with 1 T magnetic field.

The melt-spun (Nd_0.8_Ce_0.2_)_2.4_Fe_12_Co_2_B ribbons were annealed from 573 K to 1023 K for 15 min with 1 T magnetic field, see Supplementary Figure S1. The optimized magnetic properties were obtained at 773 K. Figure 1 shows magnetic hysteresis loops at room temperature for the melt-spun sample and the sample annealed at 773 K for 15 min with 1 T magnetic field. The intrinsic coercivity (*H_c_^i^*) decreases from 1285 kA∙m^−1^ for the melt-spun sample to 1189 kA∙m^−1^ for the annealed sample, whereas the remanence (*B_r_*) increases from 0.76 T to 0.82 T. As a consequence, a *(BH)_max_* with a rise of 16% (from 96 kJ∙m^−3^ to 111 kJ∙m^−3^) is obtained for the annealed sample. The key magnetic parameters are listed in Table 1. It is worth noting that the virgin magnetization curve of the melt-spun sample shows an S shape with two steps. That is, it starts with high susceptibility, followed by a lower susceptibility part, then increases to saturation. It is different from the virgin magnetization curve with a low initial susceptibility of melt-spun Nd-Fe-B ribbons and a steep virgin magnetization curve of sintered Nd-Fe-B magnets [4]. This difference indicates a more complicated coercivity mechanism. In order to clarify it, the temperature dependence of the coercivity was carried out in a range of 300–500 K.

For the case of strong pinning of domain walls developed by Gaunt [24], *H_c_^i^* as a function of temperature (*T*) is given by

(1) $${\left(\frac{H_{c}^{i}}{H_{0}}\right)}^{1/2}=1-{\left(\frac{75k_{B}T}{4bf}\right)}^{2/3}$$

where *H*_0_ is the critical field in the absence of thermal activation, *k_B_* is Boltzmann constant, 4*b* is the interaction range of a pin equating with the domain wall width, *δ_w_*, and *f* is the maximum restoring force per pin.

**Figure 2:**
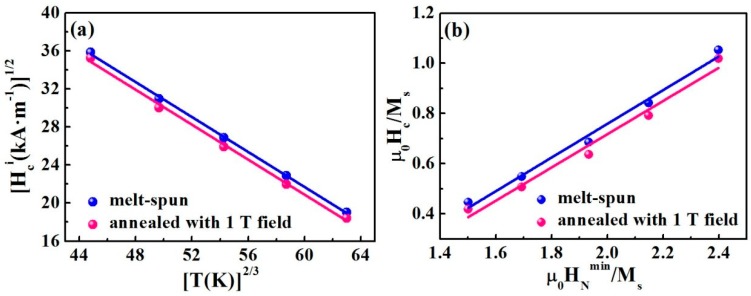
(*H_c_^i^*)^1/2^ as a function of *T*^2/3^ (**a**), and plots of *µ_0_H_c_(T)/M*_s_*(T)* versus *µ_0_H_N_^min^*(*T*)*/M_s_*(*T*) (**b**), in the temperature range of 300–500 K for melt-spun (Nd_0.8_Ce_0.2_)_2.4_Fe_12_Co_2_B ribbons and samples annealed at 773 K for 15 min with 1 T magnetic field.

The linear relationship between (*H_c_^i^*)^1/2^ and *T*^2/3^ in Figure 2a demonstrates a very good agreement with the strong pinning model in both samples. We further analyzed the temperature dependence of the coercivity by a modified form of Brown’s expression for the nucleation field [25]:

*µ*_0_*H_c_*(*T*) = *α_K_µ*_0_*H_N_^min^*(*T*) − *N_eff_M_s_*(*T*) (2)

where α*_K_* and *N_eff_* are microstructural parameters and are related to the non-ideal microstructure of a real magnet. The parameter *α_K_* describes a reduction of the nucleation field due to an inhomogeneous microstructure. *N_eff_* is an average effective local demagnetization factor. The minimum nucleation field, *µ_0_H_N_^min^*(*T*), represents a value for the nucleation field of the misaligned grains.

Equation (2) can be rewritten as

(3) $$\frac{\mu_{0}H_{c}\left(T\right)}{M_{s}\left(T\right)}=\alpha_{K}\frac{\mu_{0}H_{N}^{\min}\left(T\right)}{M_{s}\left(T\right)}-N_{eff}$$

According to Equation (3), a plot of *µ_0_H_c_*(*T*)*/M_s_*(*T*) versus *µ_0_H_N_^min^*(*T*)*/M_s_*(*T*) should yield a straight line with slope *α_K_* and intersection *N_eff_*. Figure 2b shows a linear relationship in the temperature range of 300–500 K for both samples, suggesting that the nucleation of reversed domains occurs for the magnetization reversal in the (Nd_0.8_Ce_0.2_)_2.4_Fe_12_Co_2_B alloy. The fitting parameters by a standard linear least-squares method to Equation (3), *α_K_* and *N_eff_* are shown in Table 1. The value of *α_K_* is 0.67 and 0.66, and *N_eff_* is 0.58 and 0.61 for the melt-spun sample and annealed sample, respectively. The value of *α_K_* is almost the same for both samples. The data in Figure 2 indicate that both strong pinning of domain walls and nucleation mechanisms are present in the melt-spun (Nd_0.8_Ce_0.2_)_2.4_Fe_12_Co_2_B ribbons at room temperature. Moreover, the same mechanism works in the annealed samples.

XRD patterns of the wheel surface and free surface of melt-spun (Nd_0.8_Ce_0.2_)_2.4_Fe_12_Co_2_B ribbons and annealed samples at 773 K for 15 min with 1 T magnetic field are shown in Supplementary Figure S2. The Nd_2_(Fe,Co)_14_B phase (2:14:1 phase) is observed in both samples, indicating that magnetic field annealing treatment does not change the phase constitution. Our previous work [26] found homogenous microstructure of melt-spun ribbons could be obtained by optimizing the chamber pressure and wheel speed during melt-spinning. Supplementary Figure S3a,b shows a uniform distribution of grains of cross-sectional region near the wheel surface and free surface of melt-spun (Nd_0.8_Ce_0.2_)_2.4_Fe_12_Co_2_B ribbons prepared at a wheel speed of 15 m∙s^−1^ and a chamber pressure of 0.05 MPa. The grain size distribution is determined from the TEM images, as shown in Supplementary Figure S3c,d. The average grain size is 38 ± 7 nm and 64 ± 6 nm close to the wheel surface and free surface, respectively. In comparison to the inhomogeneous microstructure with the scale of structure in a melt-spun sample varying from 100 nm to 10 µm [27], our work shows that the melt-spun (Nd_0.8_Ce_0.2_)_2.4_Fe_12_Co_2_B alloy has a homogeneous microstructure through the thickness of the ribbon. Hence, the free surface of the ribbons was chosen to further investigate the microstructure and chemical composition of the grain boundary phase.

**Figure 3:**
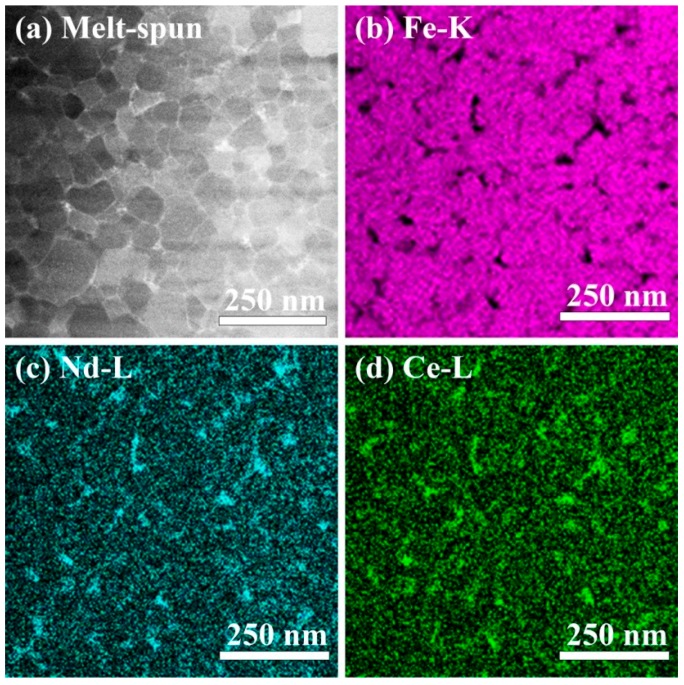
(**a**) High angle annular dark field (HAADF) image of the region close to the free surface of melt-spun (Nd_0.8_Ce_0.2_)_2.4_Fe_12_Co_2_B ribbon, and scanning transmission electron microscope with energy-dispersive X-ray spectroscopy (STEM-EDS) elemental mapping images for (**b**) Fe-K, (**c**) Nd-L and (**d**) Ce-L from the same region as (**a**).

Figure 3 shows STEM-EDS results from a region near the free surface of the melt-spun sample. In the HAADF image (Figure 3a), 2:14:1 grains surrounded by thin and continuous layers along the grain boundaries are observed. Mapping images for Fe-K, Nd-L, and Ce-L taken from the same region are shown in Figure 3b–d. Fe is depleted, whereas Nd and Ce are enriched at the GBs. The annealed sample shows a similar result, as shown in Supplementary Figure S4. In both samples, no obvious Co segregation at the GBs is observed (not shown here). Hence, APT was used for further investigations of chemical composition at the GBs.

**Figure 4:**
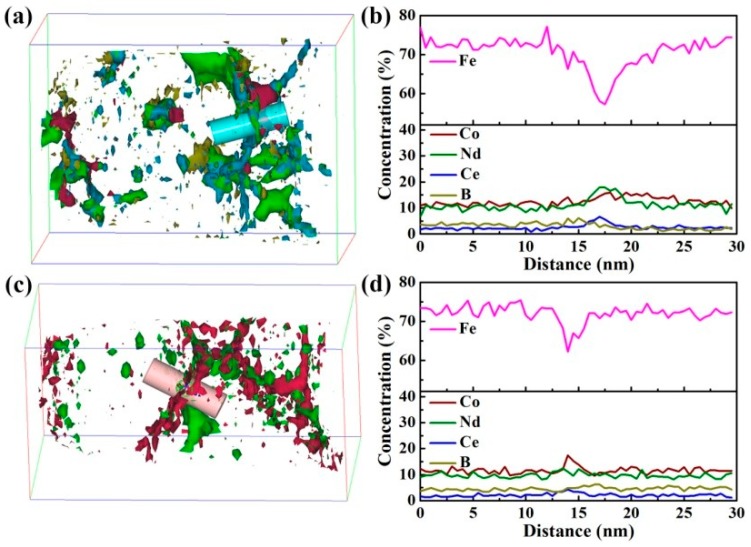
(**a**) Atom probe tomography (APT) result from the region close to the free surface of melt-spun (Nd_0.8_Ce_0.2_)_2.4_Fe_12_Co_2_B ribbons: APT reconstruction illustrates segregation by isoconcentration surfaces of 15 at% Nd (green), 4 at% Ce (blue), 14 at% Co (red), and 6 at% B (yellow); (**b**) The concentration depth profiles obtained from the selected analyzed volume shown in (**a**); (**c**) APT result from the region close to the free surface of the (Nd_0.8_Ce_0.2_)_2.4_Fe_12_Co_2_B sample annealed at 773 K for 15 min with 1 T magnetic field: APT reconstruction illustrates segregation by isoconcentration surfaces of 12 at% Nd (green) and 14 at% Co (red); (**d**) The concentration depth profiles obtained from the selected analyzed volume shown in (c).

Figure 4a,b show the APT result from the region close to the free surface of the melt-spun sample. To determine the chemical composition of the segregation at the GBs, isoconcentration surfaces of 15 at% Nd (green color), 4 at% Ce (blue color), 14 at% Co (red color), and 6 at% B (yellow color) are used (see Figure 4a). It is found that Nd and Ce are enriched at the GBs, whereas the segregation of Co and B is visible in some regions at the GBs. An analysis volume of 10 nm × 10 nm × 30 nm from Figure 4a was selected, the corresponding concentration depth profiles are shown in Figure 4b. The amounts of Nd, Ce, Fe, and Co at the GB are 18 ± 1 at%, 8 ± 1 at%, 58 ± 1 at%, and 16 ± 1 at%, respectively. Figure 4c,d show the APT results from the region near the free surface of the annealed sample with 1 T magnetic field. Isoconcentration surfaces of 12 at% Nd (green color) and 14 at% Co (red color) are used to visualize and identify the grain boundary. In comparison to the melt-spun sample (Figure 4a), Co segregation is obviously found at the GBs. The concentration depth profiles from the analysis volume of 10 nm × 10 nm × 30 nm from Figure 4c are shown in Figure 4d. The amount of Co at the GB is 18 ± 1 at%, which is higher than 16 ± 1 at% in the melt-spun sample. It indicates that magnetic field annealing increases the segregation of Co at the GBs. It is worth noting that the concentration of Fe + Co (74 ± 1 at%) and (80 ± 1 at%) is found at the GBs for the melt-spun sample and annealed sample, respectively, which are higher than 65 at% reported by Sepehri-Amin [16].

## 4. Discussion

### 4.1. Exchange Coupling Interaction through Ferromagnetic GB Phase

**Figure 5:**
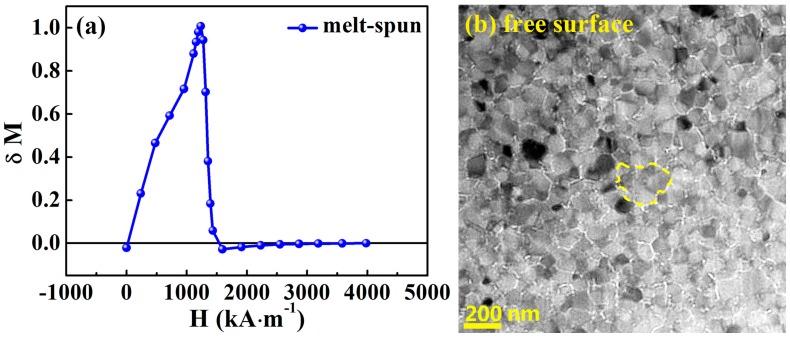
(**a**) *δM* plot as a function of applied field, and (**b**) Lorentz transmission electron microscopy (TEM) image from the region close to the free surface of the melt-spun (Nd_0.8_Ce_0.2_)_2.4_Fe_12_Co_2_B sample.

In 2012, Sepehri-Amin et al., reported that a thin GB phase with high concentration of Fe + Co (~65 at%) was observed in sintered NdFeB magnets by three-dimensional atom probe, and suggested that this thin GB phase was ferromagnetic [16]. Furthermore, the ferromagnetic GB phase in other sintered NdFeB magnets was confirmed by electron holography, soft X-ray magnetic circular dichroism (XMCD), and spin-polarized scanning electron microscopy (spin SEM), respectively [17,18,19]. In our work, the APT result (Figure 4) showed that the amount of ferromagnetic elements (Fe + Co) at the GBs of melt-spun sample and the annealed sample is 74 ± 1 at% and 80 ± 1 at%, respectively, which are higher than 65 at% reported by Sepehri-Amin [16]. It is reasonable to believe that the GB phase observed in (Nd_0.8_Ce_0.2_)_2.4_Fe_12_Co_2_B alloy is ferromagnetic, which leads to an exchange coupling interaction between the hard magnetic 2:14:1 grains. An effective method of understanding the exchange coupling interaction is via the so-called *δM* plot. Based on Wohlfarth’s theory; Kelly et al. [28] defined

(4) $$\delta M=M_{d}\left(H\right)-\left[1-2M_{r}\left(H\right)\right]$$

where *H* is an applied magnetic field, *M_d_ (H)* is the reduced magnetization, and *M_r_(H)* is the reduced remanence magnetization. The values of *M_d_(H)* and *M_r_(H)* were obtained from the measurement of the IRM and DCD curves. It is worth noting that the sample for the IRM curve was virgin state. Hence, the IRM curve of the annealed sample with 1 T magnetic field could not be measured due to its magnetized state. Figure 5a shows the *δM* plot of the melt-spun sample. The curve is initially positive, indicating the existence of exchange coupling interaction dominating the magnetization. Then the curve drops to negative values, suggesting that magnetostatic interaction is dominant. The obvious positive *δM* peak in Figure 5a confirms the existence of strong exchange coupling interaction between 2:14:1 grains. Figure 5b shows the Lorentz microscopy image of the cross-sectional region near the free surface of melt-spun ribbon. This was observed in the Fresnel mode. The size of the domain (marked as yellow dashed line) is about 290 nm, which is close to the critical size for single-domain particles (ᾂ ≈ 300 nm for Nd_2_Fe_14_B [29]). It is much larger than the average grain size of 64 nm. That is, a domain includes several grains. Such a domain is also termed an interaction domain due to magnetic coupling between neighboring grains [30]. It indicates that the hard magnetic 2:14:1 grains have an exchange coupling interaction through the ferromagnetic GB phase.

**Figure 6:**
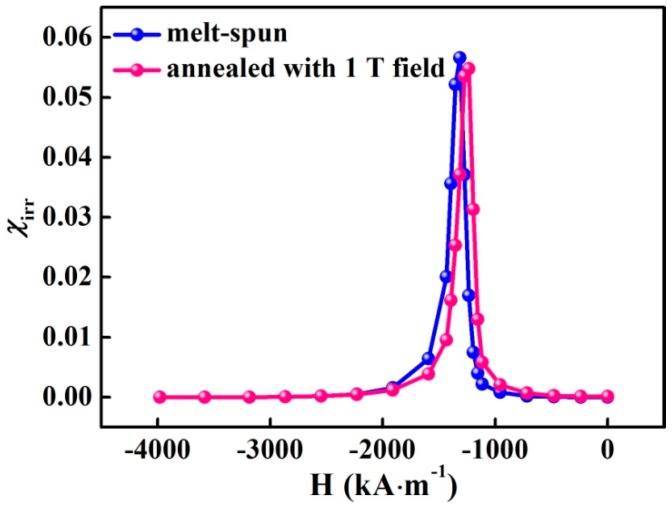
The irreversible susceptibility, *χ_irr_*, curves as a function of applied field for the melt-spun (Nd_0.8_Ce_0.2_)_2.4_Fe_12_Co_2_B sample and the sample annealed at 773 K for 15 min with 1 T magnetic field.

In the annealed sample with 1 T field, the GB phase with high concentration of ferromagnetic elements (Fe + Co = 80 ± 1 at%) enhances the exchange coupling and can be demonstrated through the irreversible susceptibility (*χ_irr_*) curve. Figure 6 shows *χ_irr_* curves for the melt-spun and annealed samples. A single sharp peak is observed in both samples, which is typical of a single-phase magnet [31]. The narrow and intensive peak in the *χ_irr_* curves indicates that each grain couples well with its neighboring grains due to exchange coupling between the magnetic phases. The annealed sample shows a narrower peak than the melt-spun sample, suggesting a stronger exchange coupling interaction through the ferromagnetic GB phase. As a consequence, the remanence is enhanced by about 8% from 0.76 T to 0.82 T.

### 4.2. The Coercivity Mechanism

In melt-spun Nd-Fe-B ribbons, the pinning of magnetic domain walls is believed to be a dominant coercivity mechanism [4]. In our work, the S-shape of the virgin magnetization curve of the melt-spun sample was observed, which is different from that of the melt-spun Nd-Fe-B ribbons (it exhibits lower initial susceptibility, reaching a higher susceptibility before saturation). Moreover, the amount of (Fe + Co) at the GBs is more than 74 at%, and the GB phase is ferromagnetic. In such a case, the previous coercivity mechanism in melt-spun Nd-Fe-B ribbons with GB phase should be reconsidered. Our results show that the coercivity of melt-spun (Nd_0.8_Ce_0.2_)_2.4_Fe_12_Co_2_B ribbons with the ferromagnetic GB phase at room temperature is from the combination effects of pinning of domain walls and nucleation. The microstructural investigations show that the 2:14:1 grains are surrounded by thin layers at the GBs, which can provide more pinning sites for domain wall motion. That is, the regions at the GBs are responsible for domain wall pinning because the presence of the ferromagnetic GB phase with lower magnetocrystalline anisotropy may give rise to a pinning force for magnetic domain wall motion. The plot of *μ_0_H_c_(T)/M_s_(T)* versus *μ_0_H_N_^min^(T)/M_s_(T)* for melt-spun (Nd_0.8_Ce_0.2_)_2.4_Fe_12_Co_2_B ribbons in Figure 2b gives the rather surprising result that Equation (3) is equally applicable to ribbons. The ferromagnetic GB phase can stimulate the nucleation of reversed domains. The parameters *α_k_* = 0.67 and *N_eff_* = 0.58 of melt-spun (Nd_0.8_Ce_0.2_)_2.4_Fe_12_Co_2_B ribbons do differ markedly from 0.25 and 0.26 observed in the melt-spun Nd-Fe-B ribbons [32]. The large value of *α_K_* and small value of *N_eff_* are helpful for the enhancement of the coercive field in the NdFeB materials [33]. In Table 1, parameter *α_K_* is 0.67 and 0.66, and is almost the same for the melt-spun sample and the annealed sample. The value of *N_eff_* is 0.58 for the melt-spun sample, which is smaller than 0.61 for the annealed sample. That is, the melt-spun sample has a larger value of the coercivity, see Table 1. The magnetic field annealing treatment enhances the remanence due to an improvement of the exchange coupling interaction through ferromagnetic GB phase. Hence, the value of (*BH*)*_max_* (111 kJ∙m^−3^) in this work for (Nd_0.8_Ce_0.2_)_2.4_Fe_12_Co_2_B ribbons annealed with 1 T magnetic field is 11% higher than 100 kJ∙m^−3^ of the same alloy prepared by melt-spinning and subsequent annealing without magnetic field [10]. Moreover, the value of (*BH*)*_max_* (111 kJ∙m^−3^) at room temperature is also 23% higher compared to 5.9 wt% Dy containing Nd-Fe-B ribbons [34].

## 5. Conclusions

In summary, we investigated the relationship between the microstructure and magnetic properties of melt-spun (Nd_0.8_Ce_0.2_)_2.4_Fe_12_Co_2_B ribbons and annealed samples at 773 K for 15 min with 1 T magnetic field. The exchange couple interaction through the ferromagnetic GB phase was confirmed by the *δ*M-H curve and Lorentz microscopy. Magnetic field annealing increases the segregation of Co leading to a concentration of (Fe + Co) with 80 ± 1 at% at the GBs, which results in a stronger exchange coupling interaction between the 2:14:1 grains. Hence, compared to the melt-spun sample, the (*BH*)*_max_* was improved by 16% after annealing with a 1 T field. We propose that the coercivity of the melt-spun (Nd_0.8_Ce_0.2_)_2.4_Fe_12_Co_2_B ribbon with ferromagnetic GB phase at room temperature is from the combined effects of the pinning of the domain walls and nucleation. Magnetic field annealing treatment does not change the coercivity mechanism. Our findings give further insight into the coercivity mechanism of melt-spun Nd-Fe-B ribbons with GB phase and provide a new idea to design low-cost and prospective permanent alloys with ferromagnetic GB phase.

## Figures and Tables

**Table 1:** The intrinsic coercivity (*H_c_^i^*), the remanence (*B_r_*), maximum energy product ((*BH*)*_max_*), the microstructural parameters *α_k_*, and *N_eff_* of melt-spun (Nd_0.8_Ce_0.2_)_2.4_Fe_12_Co_2_B sample and annealed sample at 773 K for 15 min with 1 T magnetic field.

(Nd_0.8_Ce_0.2_)_2.4_Fe_12_Co_2_B Alloy	*H_c_^i^* (kA∙m^−1^)	*B_r_* (*T*)	(*BH*)*_max_* (kJ∙m^−3^)	*α_k_*	*N_eff_*
Melt-spun sample	1285	0.76	96	0.67	0.58
Annealed sample	1189	0.82	111	0.66	0.61

## Supplementary Materials

The following are available online at www.mdpi.com/1996-1944/10/9/1062/s1. Figure S1: Magnetic properties of melt-spun (Nd_0.8_Ce_0.2_)_2.4_Fe_12_Co_2_B ribbons annealed at various temperatures with 1 T magnetic field. Figure S2: X-ray diffraction patterns of the wheel surface (a) and free surface (b) of melt-spun (Nd_0.8_Ce_0.2_)_2.4_Fe_12_Co_2_B ribbons and annealed samples at 773 K for 15 min with 1 T magnetic field. Figure S3: Bright-field TEM image of the wheel surface (a), and free surface (b); Distribution histograms of grain size of the wheel surface (c), and free surface (d) of melt-spun (Nd_0.8_Ce_0.2_)_2.4_Fe_12_Co_2_B ribbon. Figure S4: (a) HAADF image of the region close to the free surface of (Nd_0.8_Ce_0.2_)_2.4_Fe_12_Co_2_B sample annealed at 773 K for 15 min with 1 T magnetic field, and STEM-EDS elemental mapping images for (b) Fe-K, (c) Nd-L and (d) Ce-L from the same region as (a).
