# Supplementary material for: Coercivity Mechanism of (Nd0.8Ce0.2)2.4Fe12Co2B Ribbons with Ferromagnetic Grain Boundary Phase
Source: Materials (Basel). 2017 Sep 11;10(9):1062. doi: 10.3390/ma10091062 (PMC5615716; doi:10.3390/ma10091062)
Supplement: Supplementary file 1 [file materials-10-01062-s001.pdf]

Supplementary Materials: Coercivity Mechanism of $(Nd_{0.8}Ce_{0.2})_{2.4}Fe_{12}Co_{2}B$ Ribbons with Ferromagnetic Grain Boundary Phase

Heyun Li, Yang Liang, Xiaohua Tan, Hui Xu, Pengfei Hu and Kezhi Ren

Figure S1 shows that melt-spun $(Nd_{0.8}Ce_{0.2})_{2.4}Fe_{12}Co_{2}B$ ribbons are annealed from 573 K to 1023 K for 15 min with 1 T magnetic field. The optimized magnetic properties are obtained at 773 K.

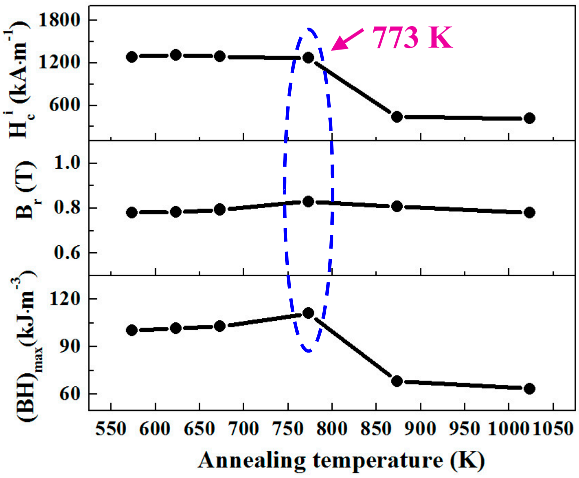

Figure S1. Magnetic properties of melt-spun $(Nd_{0.8}Ce_{0.2})_{2.4}Fe_{12}Co_{2}B$ ribbons annealed at various temperatures with 1 T magnetic field.

Figure S2 shows XRD patterns of the wheel surface and free surface of melt-spun $(Nd_{0.8}Ce_{0.2})_{2.4}Fe_{12}Co_{2}B$ ribbons and annealed samples at 773 K for 15 min with 1 T magnetic field. The $Nd_2(Fe,Co)_{14}B$ phase (2:14:1 phase) is observed in both samples.

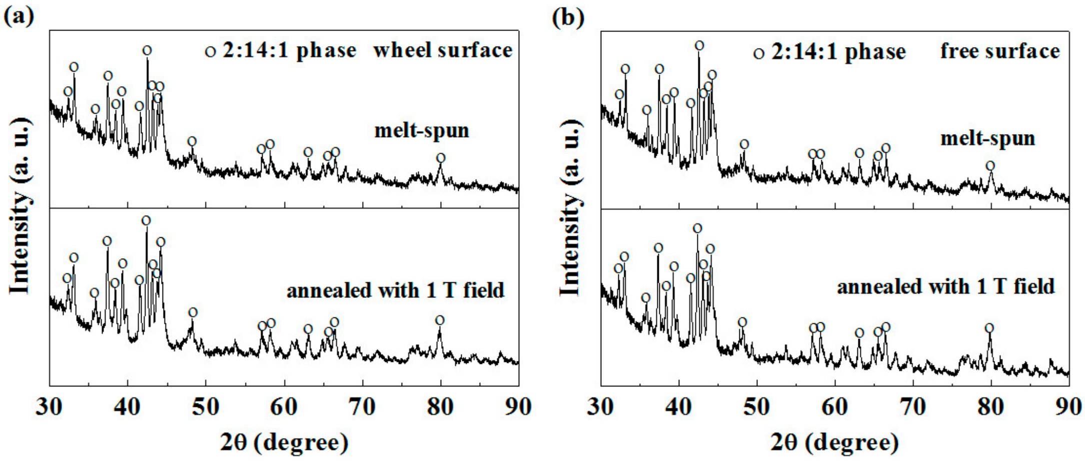

Figure S2. X-ray diffraction patterns of the wheel surface (**a**) and free surface (**b**) of melt-spun $(Nd_{0.8}Ce_{0.2})_{2.4}Fe_{12}Co_{2}B$ ribbons and annealed samples at 773 K for 15 min with 1 T magnetic field.

Figure S3 shows that melt-spun $(Nd_{0.8}Ce_{0.2})_{2.4}Fe_{12}Co_{2}B$ alloy has a homogeneous microstructure through the thickness of the ribbon. The grain size distribution is determined from the TEM images. The average grain size is 38 ± 7 nm and 64 ± 6 nm close to the wheel surface and free surface, respectively.

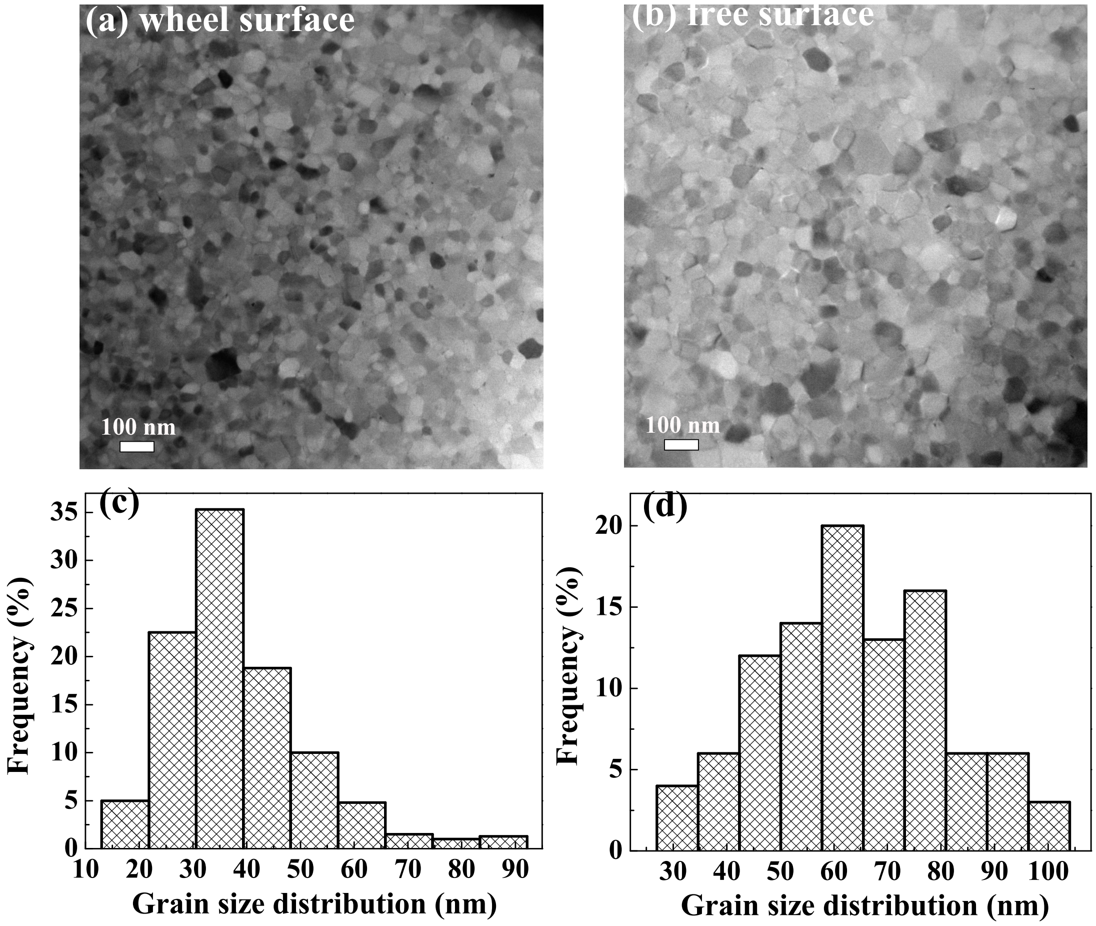

Figure S3. Bright-field TEM image of the wheel surface (**a**), and free surface (**b**); Distribution histograms of grain size of the wheel surface (**c**), and free surface (**d**) of melt-spun $(Nd_{0.8}Ce_{0.2})_{2.4}Fe_{12}Co_2B$ ribbon.

Figure S4 shows STEM-EDS result from a region near the free surface of annealed sample at 773 K for 15 min with 1 T magnetic field. HAADF image (Figure S4a) shows hard magnetic grains surrounded by thin and continuous layers along the grain boundaries (GBs). Fe is depleted, whereas Nd and Ce enrich at the GBs, see Figure S4b-d.

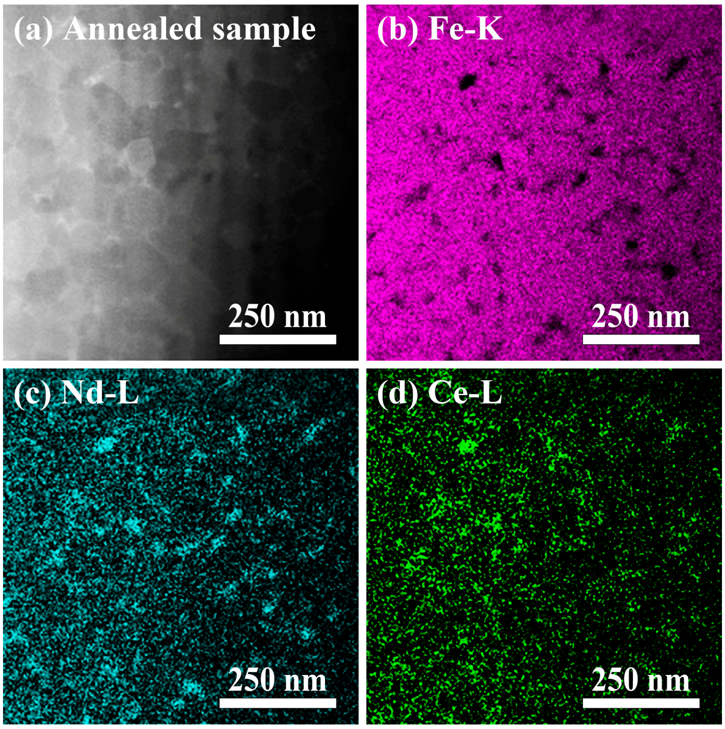

Figure S4. (**a**) HAADF image of the region close to the free surface of $(Nd_{0.8}Ce_{0.2})_{2.4}Fe_{12}Co_2B$ sample annealed at 773 K for 15 min with 1 T magnetic field, and STEM-EDS elemental mapping images for (**b**) Fe-K, (**c**) Nd-L and (**d**) Ce-L from the same region as (a).
